# Refined protocol for quantifying hippocampal neurogenesis in organotypic slice cultures

**DOI:** 10.3389/ebm.2026.11165

**Published:** 2026-09-28

**Authors:** Irem Akyel, Graham K. Sheridan

**Affiliations:** School of Life Sciences, University of Nottingham, Nottingham, United Kingdom

**Keywords:** dentate gyrus, hippocampus, neural stem cell, neurogenesis, organotypic slice culture

## Abstract

While human iPSC-derived models of the brain provide valuable genetic context, organotypic slice cultures from rodents remain essential for capturing the physiological complexity of intact neural tissue. This protocol describes a refinement of the interface method originally established by Stoppini and colleagues in 1991, providing a robust platform for the long-term cultivation of rodent hippocampal slices. Unlike dissociated monolayer systems, these 3-dimensional (3D) brain slices preserve the endogenous cytoarchitecture and the native extracellular matrix (ECM) of the central nervous system (CNS). This structural integrity is paramount for investigating hippocampal neurogenesis, as the proliferation, migration, and circuit integration of neural progenitors are governed by mechanical and biochemical cues present in the specialized local niche. By maintaining the spatial relationships between neurons, astrocytes, and microglia, this organotypic system replicates the arrangement of hippocampal neural networks more precisely than emergent stem cell models. Our protocol outlines an optimized workflow for McIlwain tissue chopper-based sectioning and maintenance of slices on porous hydrophilic membranes, ensuring efficient nutrient exchange and sustained tissue viability for several weeks. This configuration provides simultaneous access to the hippocampal slices and culture medium, facilitating the use of multiple investigative techniques such as live-cell imaging, electrophysiology, or pharmacological treatments. Despite relying predominantly on rodent tissue due to the scarcity of live human brain samples; the ability to observe developmental processes within the preserved architectural framework of the hippocampus makes this technique a cornerstone for studying neurogenesis and circuit assembly. Importantly, this refined approach bridges the gap between disorganised monolayers of *in vitro* neural networks and complex *in vivo* studies, offering accessible and reproducible physiological models to interrogate biochemical enhancers of neurogenesis or the pathological mechanisms of neurodevelopmental disorders.

## Impact statement

Organotypic hippocampal slice cultures are a vital ex vivo model of the brain because they preserve the three‑dimensional structure, native cell types, and local microenvironment that shape how new neurons are born, migrate, and integrate into neural networks. This protocol provides a simple, reproducible workflow to keep intact rodent hippocampal tissue healthy for weeks, enabling simultaneous live imaging, electrical recording, or targeted pharmacological treatments. It advances the field by combining a short, low‑serum recovery period with a defined supplement regimen to balance immediate trophic support and long‑term cellular specificity. The hippocampal slices retain the neurogenic niche that governs progenitor cell behaviour and circuit maturation, as well as the native extracellular matrix scaffold such as perineuronal net structures; brain tissue features that current stem cell models rarely recapitulate. As such, this protocol offers a practical, high‑fidelity platform that complements human iPSC models and strengthens translational studies of development, plasticity, and repair.

## Introduction

The hippocampus is an important and unique brain region that contains a specialized neurogenic niche within the subgranular zone (SGZ) of the dentate gyrus (DG) where neurogenesis continues throughout postnatal development and adulthood, supporting structural and functional plasticity mechanisms that regulate lifelong learning and memory formation. Postnatal hippocampal neurogenesis is a finely tuned physiological process with distinct stages controlled by molecular, biochemical and mechanical cues [1, 2]. Neurogenesis begins when neural stem cells (NSCs) in the SGZ divide asymmetrically, resulting in both the maintenance of the stem cell reservoir and production of nascent progenitor cells which can differentiate into newborn neurons [3]. Through tightly regulated activity-dependent events, neurogenesis fundamentally contributes to brain development, maturation and hippocampal circuit adaptability, highlighting the importance of maintaining a healthy population of neural stem cells [4–8].

Organotypic hippocampal slice cultures (OHSC) offer a powerful *ex vivo* model to study neurogenesis, neural circuit function, and the cellular microenvironment of brain tissue in a reproducible and controlled setting that closely mimics *in vivo* conditions [9]. The term *organotypic* refers to the retention of the tissue’s original properties, including its cytoarchitecture and extracellular environment. Therefore, organotypic brain slice cultures closely recapitulate the *in vivo* state by preserving the basic connective and structural tissue organization and complex neural circuitry [10].

Organotypic culturing methods have evolved considerably over the past several decades. Early approaches used the roller tube technique in which slices were mounted on coverslips and rotated within glass tubes to enhance oxygenation [11]. Although foundational, the approach was low throughput and technically demanding. The introduction of the interface method by Stoppini and colleagues [12], which utilised 0.4 µm porous hydrophilic transparent membrane inserts, provided a major advancement. Slices could now be maintained for weeks to months in culture at the air–liquid interface while preserving long-term viability and *in vivo*-like cytoarchitecture of the hippocampus. This approach rapidly became a standard technique for studies of hippocampal development, synaptic plasticity, and neural circuitry [10, 13–17].

Here, we present a comprehensive protocol for preparing and culturing postnatal day 7–10 mouse organotypic hippocampal slices on membrane inserts. Notably, the protocol outlined herein offers a streamlined procedure, achievable within a modest timeframe of 3 h, and is adaptable across developmental stages, rodent species, and brain regions [18]. The method offers direct access to hippocampal slices, enabling genetic manipulations, biochemical assays, live-cell imaging, drug treatment, electrophysiological experiments, and a wide range of functional investigations. By combining the structural and functional fidelity of *in vivo* models with the accessibility of *in vitro* systems, this protocol offers a versatile and robust platform for investigating hippocampal neurogenesis and related aspects of brain plasticity.

## Materials and methods

### Laboratory equipment

- McIlwain Tissue Chopper (Campden Instruments, Model TC752)
- Stereomicroscope (required for separating hippocampal slices after chopping)
- Class II laminar flow safety cabinet
- 5% CO_2_ incubator maintained at 35.5°C

### Dissection tools

- Standard Surgical Scissors (Fine Science Tools; item no. 14002-13)
- Fine Iris Scissors (Fine Science Tools; item No. 14060-10)
- Angled Dissecting Scissors (Fine Science Tools; Item No. 14082-09)
- Dissecting tweezers
- Blunt rounded spatula
- Short blunt forceps
- Fine curved forceps
- Scalpel and blades
- Stainless steel double-edge razor blades for McIlwain tissue chopper (Gillette)
- 27 Gauge 0.4 × 13 mm sterile syringe needles

### Slice preparation

- Sterile Millicell 6-well culture plate inserts (Millipore, cat. no. PICM0RG50)
- Sterile 6-well cell culture plates (Greiner, Bio-One six-well multi dishes, cat. no. 657160)
- Petri dishes–diameter 35 mm (CytoOne cat. no. CC7672-3340)
- Petri dishes–diameter 60 mm (CytoOne cat. no. CC7672-3359)
- Petri dishes–diameter 100 mm (CytoOne cat. no. CC7672-3394)
- Whatman Grade 1 Filter Paper, circle, 90 mm
- Disposable sterile plastic Pasteur pipettes (3 mL)
- Parafilm
- Sterile 50 mL and 15 mL Falcon tubes
- 70% ethanol

### Culture media and reagents

- Opti-MEM™ (1×) [+Glutamax, +HEPES, +2.4 g/L Sodium Bicarbonate] (Gibco-Invitrogen cat. no. 51985-034)
- DMEM/F-12 [+2.5 mM L-Glutamine, +15 mM HEPES, +110 mg/L Sodium Pyruvate, sterile-filtered] (cat. no. Cytiva- SH30261.01)
- Neurobasal-A medium (1×) [(−) L-Glutamine] (Gibco-Invitrogen, cat. no. 10888-022)
- Leibovitz’s L-15 Medium (Thermofisher Scientific cat. no. 11415064)
- Phosphate buffered saline (PBS)
- Glutamax (100×) (Gibco-Invitrogen, cat. no. 35050-038)
- D-glucose (45% solution) (Sigma, cat. no. G8769-100 ML)
- Horse serum, sterile filtered (Sigma, cat. no. H1270)
- Foetal Bovine Serum (FBS), sterile filtered (Sigma, cat. no. S0615)
- NGF (Nerve Growth Factor) (Sigma, N8133)
- MITO+ serum extender (Corning, 355006)
- N21 supplement (Millipore, SCM081)
- B27 supplement (Gibco, 17504-44)

### Reagent preparation

- Leibovitz’s L-15 medium is used as dissection and tissue chopping medium. Store in 50 mL aliquots at +4 °C.
- Nerve growth factor (NGF): Dissolve 0.1 mg in 1 mL of sterile distilled water to prepare a stock concentration of 100 μg/mL. Store in 10 µL aliquots at −20°C. Use at a final concentration of 10 ng/mL NGF in slice culture medium.
- MITO+ serum extender (dissolve lyophilized powder according to manufacturer’s instructions). Store in 25 µL aliquots at −20 °C.
- Horse serum for slice culture medium. Store in 10 mL aliquots at −20 °C.
- Foetal bovine serum (FBS) for slice culture medium. Store in 10 mL aliquots at −20 °C.
- CRITICAL STEP: Add the appropriate volumes and final concentrations of MITO+ serum extender and NGF stock solutions to the slice culture media on the day of the media change.
- CRITICAL STEP: Warm and equilibrate the slice culture media to 35.5 °C in the 5% CO_2_ incubator before transferring hippocampal slices to fresh media.

### Procedure for preparing 100 mL of organotypic slice culture media

Autoclave and sterilise two 100 mL Duran™ glass bottles and allow to cool down. Spray the outer surface of the bottles with 70% ethanol and transfer to the laminar flow safety cabinet. Prepare the slice culture media under aseptic conditions. Referring to Table 1, add the components listed for Media #1 and Media #2 to separate 100 mL bottles and mix gently. The media can then be poured into 2 × 50 mL Greiner™ centrifuge tubes (sterile), sealed, wrapped in parafilm and transferred to the fridge and stored at 4 °C for one to two weeks. Once re-opened, use within 1 week. It is advisable to prepare small volume batches of media rather than larger volumes that are opened more often.

**TABLE 1:** Formulation of culture medium #1 and culture medium #2 for organotypic hippocampal slices.

Media #1 composition Days 0–3	Volume (mL) per ∼100 mL	D-glucose (mM) per ∼100 mL
DMEM/F12 (with L-glutamine, HEPES, and sodium pyruvate)	**65**	9.1
Opti-MEM (with Glutamax, HEPES, and sodium bicarbonate)	**25**	1.4
Horse serum	**6**	-
Foetal bovine serum	**4**	-
D-glucose (45% solution)	**0.7**	17.5

Growth factors (add on the day)	Dilution factor	Stock conc.
MITO+ serum extender (EGF/FGF)	**1 : 1,000**	1000×
Nerve growth factor (NGF)	**1 : 10,000**	100 μg/mL

Media #2 composition Day 3 onwards	Volume (mL) per ∼100 mL	D-Glucose (mM) per ∼100 mL
Neurobasal-A (with HEPES, without L-glutamine)	**93**	23.3
Horse serum	**3**	-
Foetal bovine serum	**2**	-
Glutamax (100×)	**1**	-
D-glucose (45% solution)	**0.2**	5.0

Supplements	Volume (mL)	Stock conc.
B-27	**0.7**	50×
N21	**0.7**	50×

Growth factors (add on the day)	Dilution factor	Stock conc.
MITO+ serum extender (EGF/FGF)	**1 : 1,000**	1000×
Nerve growth factor (NGF)	**1 : 10,000**	100 μg/mL

### Media change procedure for organotypic slice cultures

- On Culture Day 0, add 1.1 mL of Media #1 to each well of the 6-well plate. Media can be cold when positioning freshly chopped slices onto Millicell inserts. Transfer the 6-well plates to a warm humidified CO_2_ incubator.
- On Culture Day 1, remove old media and add 1.1 mL of fresh Media #1 to each well of the 6-well plate. Pre-warm the 1.1 mL of fresh culture medium before transferring slices.
- On Culture Day 3, mix 0.55 mL of Medium #1 with 0.55 mL of Medium #2 in each well of the 6-well plate. Pre-warm the media in the incubator before transferring slices.
- On Culture Day 5, completely switch to Medium #2.
- From day 5 until the experiment end date, media changes should be performed every 48 h to avoid excess evaporation and to maintain slice health.

## Experimental procedure

### Preparing for the dissection

- Timing: 20 min
  1. Clean and sterilise the laminar flow hood with 70% ethanol.
  2. Clean and sterilise the dissection tools with 70% ethanol.
  3. Attach a double-edged blade (sterilized with 70% ethanol and allowed to dry) to the McIlwain tissue chopper.
  4. Pour 50 mL of Leibovitz’s L-15 medium into a 50 mL Falcon tube and then seal with parafilm and keep on ice.
  5. Fill a 60 mm Petri dish with 5 mL of L-15 medium and keep on ice. This can be used to store the second brain hemisphere whilst the first is being dissected.

### Dissection and tissue slicing

- Timing: 30 min
  6. Using 70% ethanol, gently swab the skin around the neck of the mouse pup (postnatal day 7 CD1 mice in this instance).
  7. Humanely sacrifice the animal by adhering to local guidelines and approved schedule 1 regulations.
  8. Using a sterile fine iris scissors, cut the skin along the midline of the scalp (from posterior to anterior) toward the frontal lobe and terminating past the olfactory bulbs.
  9. Trim any excess tissue at the base of the skull to expose the cerebellar region.
  10. Using an angled dissecting scissors, snip the skull covering the cerebellum and peel away the skull using fine curved forceps.
  11. Using an angled scissors, cut the skull bilaterally along the midline from caudal to rostral.

CRITICAL STEP: Ensure the tips of the scissors and forceps do not touch the brain to prevent any damage to the brain tissue.

12. Using a curved pointed forceps, peel apart the skull to expose the brain.
13. Once the skull is exposed, take a small blunt spatula and manoeuvre it under the brain from the front of the olfactory bulbs. Detach and sever the cranial nerves and scoop out the brain from the skull.
14. Place the brain into the 60 mm Petri dish containing L-15 medium using a short, rounded spatula.
15. Remove the cerebellum and then cut the brain in half along the midline using a sterilised scalpel blade.
16. Fill a 100 mm Petri dish with crushed ice and place the lid on. Place a sheet of 90 mm circular Whatman filter paper onto the lid of the Petri dish and pour a few drops of slicing medium to wet the filter paper. Transfer the brain hemisphere to the filter paper.
17. Insert the small blunt spatula below the corpus callosum and gently pull out the thalamus, septum, and underlying striatum to reveal the hippocampus. Using a scalpel, cut diagonally at a 45° angle below the hippocampus.
18. Place the hippocampus into the L-15 medium being kept on ice.
19. Using a forceps, then transfer the hippocampal brain tissue onto the white circular plastic chopping discs and position onto the McIlwain tissue chopper. Align the hippocampus perpendicular to the cutting blade.
20. Dab and absorb the excess L-15 medium with filter paper, without touching the tissue, and set the tissue chopper to cut 400 µm sections. One P7 mouse hippocampus generates, on average, ten hippocampal slices (Figure 1A).

CRITICAL STEP: The brain tissue should not be allowed to dry out; hence steps 19 and 20 should be performed quickly.

21. Using a sterile Pasteur pipette, transfer the 400 µm hippocampal slices into a 60 mm Petri dish filled with ice-cold L-15 medium.
22. Under the stereomicroscope, find the hippocampus and separate the hippocampal slices one-by-one using two 27 Gauge 0.4 × 13 mm sterile syringe needles.
23. Select and transfer, using a sterile Pasteur pipette, the non-damaged slices (i.e., no rips or tears) into a separate 100 mm Petri dish filled with ice-cold L-15 medium. Keep this dish on ice until all the hippocampal slices have been collected (Figures 1B–D).

**FIGURE 1:**
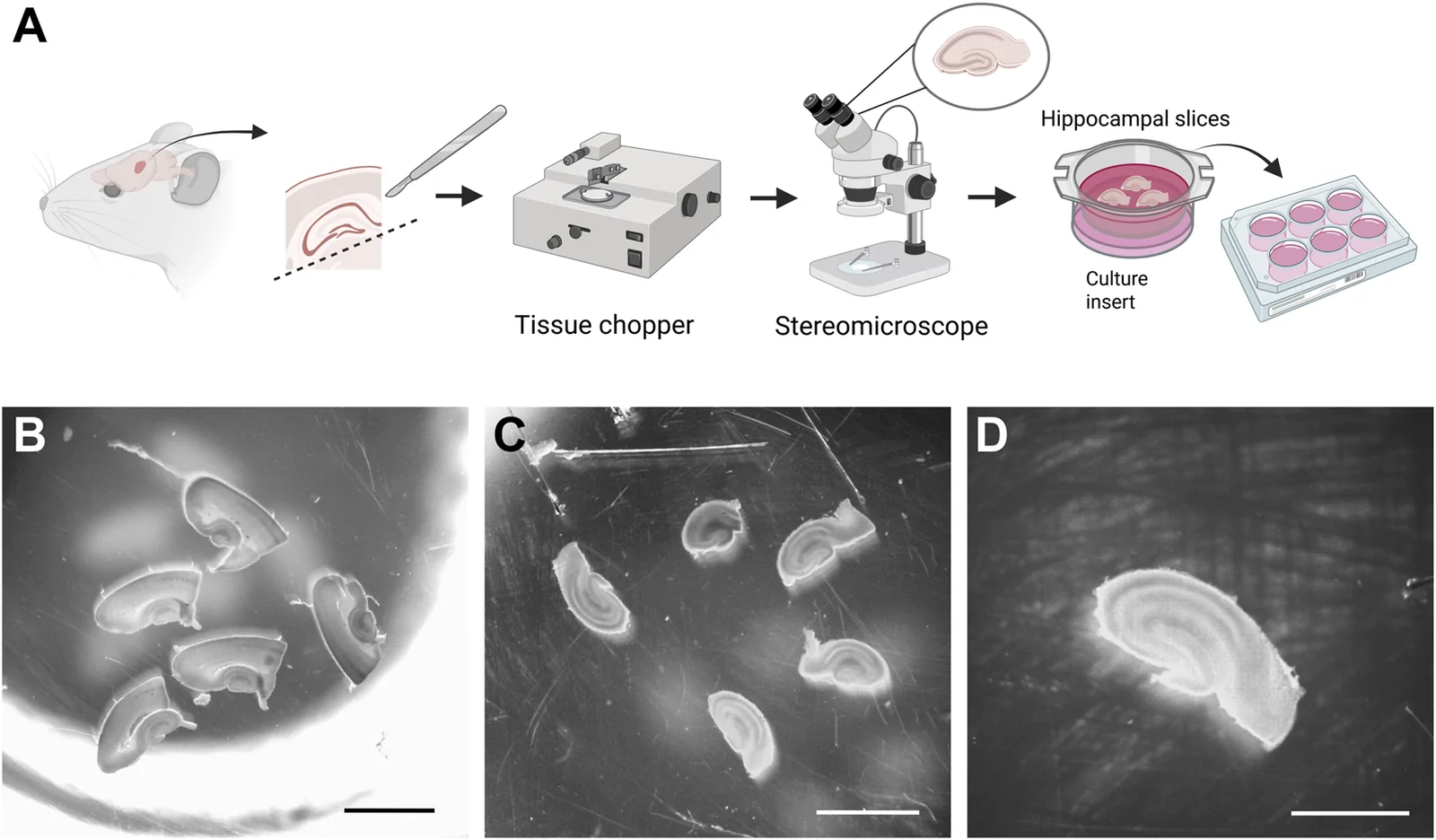
Schematic diagram of the procedure for preparing hippocampal slice cultures. **(A)** Illustration of hippocampal dissection, tissue chopping, slice separation, and culture of slices on membrane inserts in standard 6-well plates. **(B)** Separation and isolation of healthy hippocampal slices for organotypic culturing. 400 μm brain slices containing the hippocampal formation with the neocortex attached. Scale bar = 3 mm. **(C)** Hippocampal slices after the neocortex was removed with a scalpel blade. Scale bar = 2 mm. **(D)** Selected healthy and intact hippocampal slices with the neocortex removed and ready for transfer onto the culture inserts. Scale bar = 1 mm. Figure 1A created using BioRender.com.

CRITICAL STEP: Make sure the brain tissue is fully intact with no damage during separation process.

### Preparation of the 6-well culture plate

- Timing: 5 min
  24. Remove the lid of the 6-well plate and place 1.1 mL of slice culture medium #1 (with growth factors and MITO+ serum extender) into each well (see Table 1).

### Plating the hippocampal slices

- Timing: 15 min
  25. Transfer the Petri dish containing the hippocampal slices into the laminar flow cabinet.
  26. Pipette 1 mL of culture media #1 into a sterile 35 mm dish.
  27. Open the Millicell culture insert package and place an insert into the 35 mm dish using sterile forceps. The culture medium will wet the insert membrane.
  28. Take a good slice from the dish using a sterile wide-end Pasteur pipette.
  29. To plate, carefully squeeze until one drop of solution containing the slice is in the centre of the insert. Utilizing a sterile 200 µL pipette tip, remove the excess L-15 medium from the insert. The slice should lie flat on the insert.
  30. Repeat and place a total of three hippocampal slices onto the insert one-by-one using a sterile Pasteur pipette (Figure 2).

CRITICAL STEP: Ensure that none of the hippocampal slices are in contact with each other and avoid placing slices at the edge of the insert to maximise culture growth and slice health.

**FIGURE 2:**
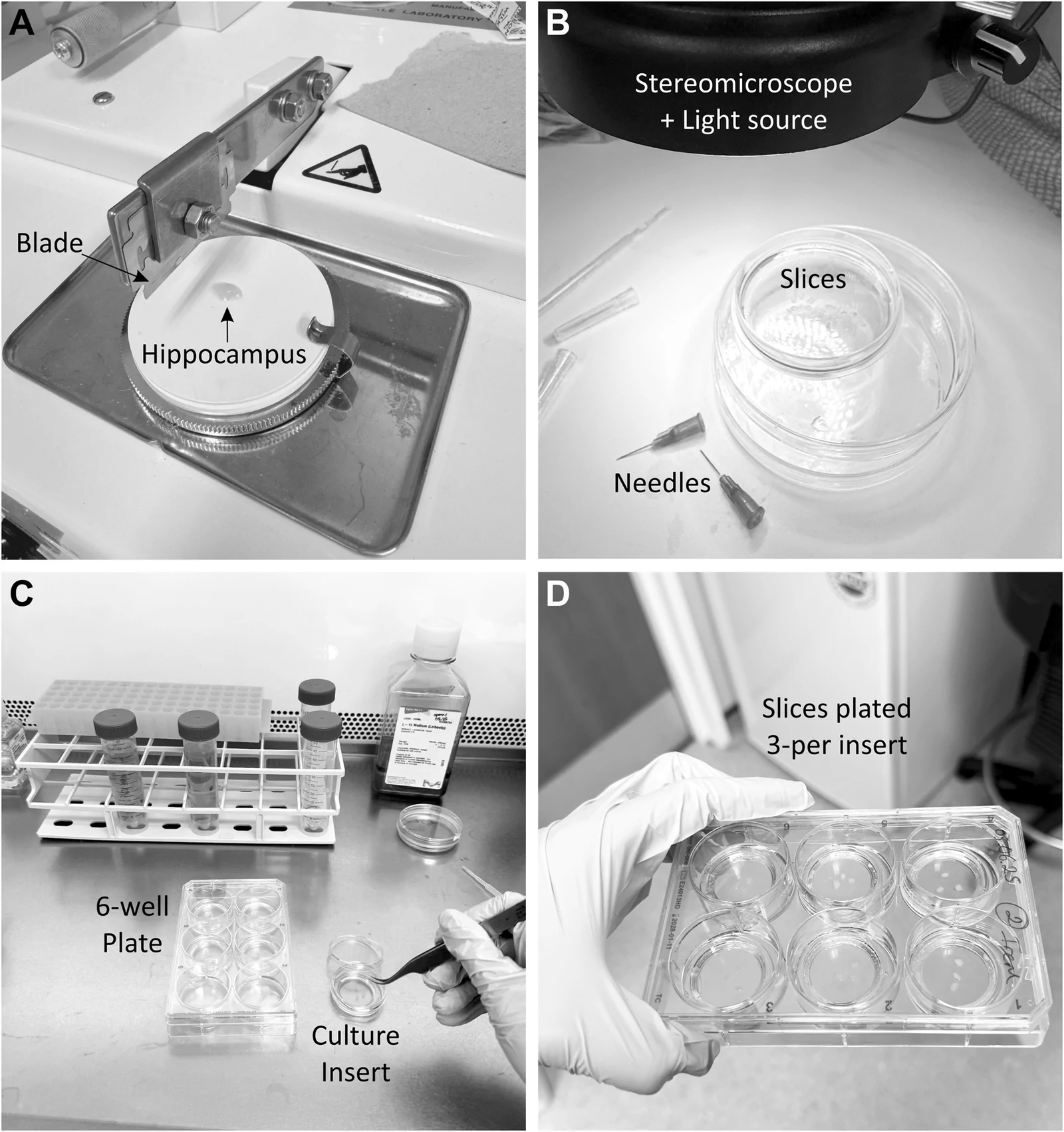
Hippocampal slice preparation steps. **(A)** Chopping of hippocampal tissue into 400 μm thick slices with the McIlwain tissue chopper. **(B)** Separation of brain slices using 27 gauge needles under an illuminated stereomicroscope. **(C)** Positioning hippocampal slices onto Millicell 0.4 µm porous membrane inserts. **(D)** Organotypic hippocampal slices housed in a 6-well plate in culture medium.

CRITICAL STEP: Make sure that the insert membrane is fully wet and free of air bubbles underneath. Record the date and culture information on the 6-well plate lid using a black marker.

31. Place a culture insert, with 3 hippocampal slices, in each well of the 6-well plate (Figure 3). Repeat for the other 5 inserts.
32. Once all the slices have been plated, transfer the 6-well plate into the 35.5 °C incubator (with 5% CO_2_).

**FIGURE 3:**
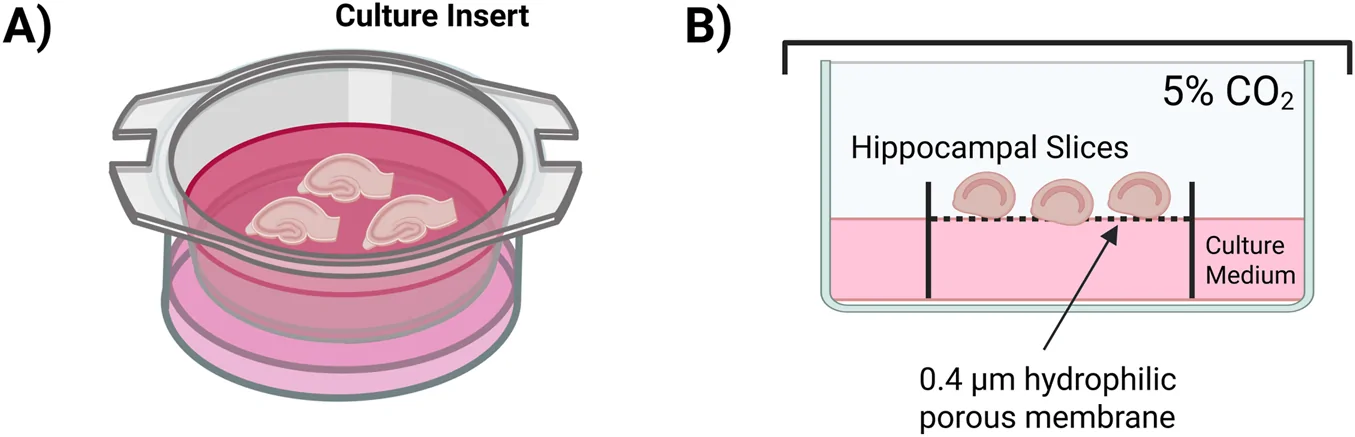
Schematic diagram of the air–liquid interface method for culturing brain slices. **(A)** 3D isometric view of a Millicell insert with three hippocampal slices. **(B)** Side-view of the culture insert incubated at 35.5 °C in a humidified atmosphere of air and 5% CO_2_. The porous membrane allows nutrients and growth factors to bathe the undersurface of the slice and the air interface promotes flattening and attachment of the slice to the hydrophilic transparent membrane. Figure created using BioRender.com.

### Changing the medium and maintaining the cultures

- Timing: 15 min
  33. The culture medium should be changed on Day 1 (i.e. 24 h after slices are first plated) and then every 2 days after that (i.e., Days 3, 5, 7 etc. See Table 1).

CRITICAL STEP: Media changes should be completed under sterile conditions in a laminar flow cabinet.

CRITICAL STEP: Antibiotics are not used in the culture media to minimize cytotoxicity and to improve tissue viability. Therefore, a high standard of aseptic technique and sterility is critical.

34. Based on the number of wells, calculate the volume of culture medium required (i.e. 1.1 mL per well plus 1 mL extra) and pipette into a Falcon tube.
35. Add the NGF and MITO+ serum extender into the media using a sterile pipette.
36. Mix and pipette the complete media into the wells of a 6-well plate and then place the plate into the humidified CO_2_ incubator.

NOTE: Warming the medium to 35.5 °C in the incubator should be performed 1–2 h ahead of time.

37. Transfer the 6-well plate with slices, and the new plate with fresh medium, from the incubator into the laminar flow hood.
38. Using 70% ethanol, sterilize a pair of pointed forceps and transfer the inserts from old to new medium.
39. Remove the old medium using a 1 mL pipette.
40. Using the sterile forceps, transfer each insert (by holding the plastic edge) to a new well in the 6-well plate with the pre-warmed media. Remove and dispose of the old medium from the wells. Perform the same procedure for all six wells.

CRITICAL STEP: Ensure no air bubbles are trapped under the insert.

41 Slices can be maintained and cultured for several weeks after plating. Four to 6 days after plating, the slices will be firmly attached to the insert. There is no need to coat the membrane with poly-D-lysine, poly-L-ornithine, or extracellular matrix.

#### Comparison of Media #1 versus Media #2

The transition from a DMEM/F12 + Opti-MEM mixture (Media #1) to a Neurobasal-A backbone (Media #2) represents a transition from a mitogenic environment to one optimized for the physiological maturity of 3D neural circuits (Table 2). While the original Stoppini et al., [12] method relied on a standard Hank’s balanced salt solution (HBSS)-based medium, modern refinements utilize Neurobasal-A specifically because it is formulated to support the survival of post-mitotic neurons while suppressing the overgrowth of non-neuronal cells [19] (see Tables 3, 4).

**TABLE 2:** Comparison of DMEM/F12 + OptiMEM versus Neurobasal-A media formulations.

Feature	Media #1 (early recovery)	Media #2 (maintenance phase)
Primary Goal	Recovery & attachment: Higher serum concentration to “seal” damaged cell membranes and promote slice adherence to membrane	Differentiation & health: Optimized for neuronal maturation and neurogenesis quantification
Serum content	10% total: (6% horse, 4% FBS). Rich in growth factors but its undefined composition can introduce variability	5% total: (3% horse, 2% FBS). Shifts reliance toward the defined supplements (B27/N21) while retaining low levels of growth-promoting serum components
Osmolarity	Higher (∼320–340 mOsm/L): High glucose and serum protein levels increase the osmotic pressure	Moderate (∼290–310 mOsm/L): Designed to be closer to physiological cerebrospinal fluid
Glutamate levels	Higher: Contributed by DMEM/F12 and serum. Promotes excitability and connectivity of damaged neural networks. Risk of mild excitotoxicity	Lower: Neurobasal-A is formulated to minimize glutamate to reduce excess Ca^2+^ influx and cell death. Low levels still present due to serum
Buffering (HEPES)	Higher (∼15 mM). Provides more pH stability. More metabolic waste in the first 72 h	Moderate (∼10 mM): Sufficient for stable 48-h media cycles in a CO_2_ incubator
Vitamin/Mineral profile	Broad & Diverse: DMEM/F12 provides a large range of salts and fat-soluble vitamins for general cell health	Targeted: Neurobasal-A removes the excitatory amino acids glutamate and aspartate present in DMEM/F12 to reduce excitotoxicity and improve long-term maintenance of neurons *ex vivo*
Lipid composition	Variable: Dependent on serum batches	More consistent: B27 and N21 provide defined antioxidants and a standardized mixture of lipids, including essential fatty acids (e.g., linoleic acid)
Impact on neurogenesis	Promotes proliferation of progenitor cells due to high serum growth factors	Promotes differentiation and integration of new neurons; better for quantifying mature markers

**TABLE 3:** Comparison of B27, N21 and MITO+ serum extender components.

Component category	B-27 (classic)	N21 (optimized)	MITO+ serum extender	Function in brain slice culture assay
Carrier proteins	BSA (fatty acid-free)	BSA (fatty acid-free)	Low Present in ECGS*	Binds toxins (e.g., ammonia) and stabilizes hydrophobic lipids in the media
Iron Delivery	Transferrin (human recombinant)	Holo-transferrin (bovine)	Holo-transferrin (human plasma)	Vital for mitochondrial respiration; N21/MITO+ guarantee iron-saturation, preventing the “iron-stealing” effect of Apo-transferrin
Vitamins	Biotin, Vitamin E (×2), Vitamin A (Acetate)	Biotin, Vitamin E (×2), Vitamin A (Acetate) & Retinol	Biotin only	Vitamin A (Retinoids) directs neural stem cell fate to neuronal lineage; vitamin E prevents lipid peroxidation in the hippocampal tissue
Growth factors	None	None	EGF (mouse recombinant) ECGS (FGF-1 & FGF-2)	Directly drives the cell cycle of neural stem cells to increase the pool of dividing cells
Metabolic hormones	Insulin, Corticosterone, T3, Progesterone	Insulin, Corticosterone, T3, Progesterone	Insulin, Hydrocortisone, T3, Progesterone, Testosterone, Oestradiol	Insulin drives glucose uptake; the expanded steroid suite in MITO+ provides potent mitogenic and anti-inflammatory signals
Antioxidants	Reduced Glutathione (GSH), Selenium	Reduced Glutathione (GSH), Selenium, α-Lipoic (Thioctic) acid	Lower GSH (stabilizer), Selenium	Prevents ferroptosis; Thioctic acid in N21 specifically recycles GSH, making it more effective for long-term (21+ days) slices
Antioxidant Enzymes	Catalase, Superoxide dismutase (SOD)	Catalase, Superoxide dismutase (SOD)	None	Enzymes that physically dismantle H2O2 and superoxide generated by the trauma of the slicing procedure
Lipids & precursors	Linoleic & Linolenic acids	Linoleic & Linolenic acids	o-Phosphoryl-ethanolamine	Provides the building blocks for the phospholipid bilayer of expanding dendrites in newborn neurons
Small molecules	Ethanolamine, L-Carnitine, Putrescine, Galactose	Ethanolamine, L-Carnitine, Putrescine, Galactose	None	Putrescine is a polyamine essential for DNA synthesis; L-Carnitine is required for fatty acid transport into mitochondria

* ECGS (Endothelial Cell Growth Supplement) is a specialized crude extract derived from bovine hypothalamus and contains a concentrated source of heparin-binding growth factors, primarily acidic and basic Fibroblast Growth Factors (FGF-1 and FGF-2).

**TABLE 4:** Comparison of Horse serum and Foetal Bovine serum.

Category	Foetal bovine serum (FBS)	Horse serum (HS)	Function in brain slice culture assay
Growth factors	High & Diverse: Rich in EGF, FGF, PDGF, and IGF-1	Lower: Generally, contains fewer mitogenic factors	Neurogenesis: FBS growth factors are the primary triggers for progenitor cell proliferation
Proteins	High Fetuin: Promotes cell attachment and spreading	High Albumin: Often higher total protein content than FBS	Architecture: HS is superior for promoting the “flattening” and structural integrity of the slice
Lipids & hormones	Lower Steroids: More balanced for general growth	Higher Steroids: Rich in hormones like progesterone, oestradiol and cortisol/corticosterone	Neuroprotection: Steroid hormones provide robust protection against the trauma of the slicing process
Enzyme profile	Low Cholinesterases	High Butyrylcholinesterase	Excitability: HS dampens excess cholinergic signalling, preventing excitotoxic “shock” during the recovery phase
Gamma-globulins	Minimal: Nearly absent in foetal blood	Significant: Present as part of the adult immune profile	Differentiation: Gamma-globulins can act as weak growth inhibitors, which helps “calm” the slice after the initial FBS-driven growth spurt
Immunoglobulins	Very low: Because the foetus hasn’t been exposed to pathogens	High: Adult horses have high levels of IgG and other antibodies	Compatibility: Low Ig levels prevent immune interference; high Ig can occasionally inhibit neurite outgrowth

### Neurogenesis assay (EdU protocol)

ThermoFisher Scientific catalogue number C10638

- Timing: 30 min

### Preparing stock solutions

42. Allow the vials to warm to room temperature before opening.
43. Prepare a 10 mM stock solution of EdU by adding 2 mL of DMSO.
44. Prepare a working solution of the Alexa Fluor® 555 azide by adding 70 µL of DMSO.
45. To make a 10× stock solution of the Click-iT® EdU buffer additive: Add 2 mL of deionized water to the vial, then mix until fully dissolved. Store any remaining stock solutions at −20 °C.

### Click-iT EdU cell proliferation assay

- Timing: 1 day
  46. Prepare a working solution of 5 µM EdU in slice culture media #2.
  47. Add 1.1 mL of this EdU solution to 6-well plates and pre-warm the media.
  48. Treat the slices with 5 µM EdU on the 6^th^ DIV and incubate for 6 h.
  49. Wash the inserts with PBS for approximately 30 s in 35 mL petri dishes to remove any residual EdU solution.
  50. Replace the media and culture the slices for a further 13 days.
  51. Perform fixation and permeabilization steps as described below (steps 57–70).
  52. Prepare 1× Click-iT® EdU buffer additive and prepare the Click-iT® reaction cocktail as guided (here we added 450 µL of solution per hippocampal slice in a 12-well plate) and use within 15 min of preparation.
  53. Incubate the 6-well plate for 4 h at room temperature and protect from light.
  54. Wash 3 times for 10 min with PBS.
  55. Remove the wash solution.
  56. For immunofluorescence antibody labelling, follow steps 70–81 below.

### Characterisation of hippocampal slice cultures by immunofluorescence

- Timing: 1 week

CRITICAL STEP: Perform fixation under a fume hood and wear appropriate personal protective equipment. Formalin solution is carcinogenic.

57. Prepare the fixing solution (i.e. 10% formalin in PBS) and cool on ice 15 min before fixation.
58. Place the 6-well plate on ice, aspirate and discard residual medium.
59. Add 1 mL of fixing solution above the insert and 1 mL below the insert.
60. Incubate for 10 min on ice.
61. Aspirate and remove the fixing solution and replace with 1 mL of PBS using a Pasteur pipette.
62. Incubate for 10 min with PBS at room temperature.

NOTE: To enhance visualization of the slices, perform these fixation steps on a dark background.

63. Aspirate and remove the PBS.
64. Repeat the PBS wash steps two more times.
65. Prepare the permeabilization and blocking solution containing PBS + 0.5% Triton X + 10% BSA (Bovine Serum Albumin).
66. Add 1 mL of permeabilization and blocking solution above and below each insert.
67. Seal the 6-well plate with parafilm, place on a gentle rocker, and incubate at 4°C overnight.

NOTE: Examine the hippocampal slices under the microscope to assess their structural integrity. Immunofluorescence should only be performed on slices that display a well-preserved cytoarchitecture.

CRITICAL STEP: Exercise caution throughout all pipetting steps as the hippocampal slices can detach from the insert. To avoid this, gently apply each solution drop by drop at the edge of the insert away from the slices.

68. Once the permeabilization and blocking step is complete, use a sterile scalpel to cut the membranes from the Millicell inserts to obtain a smaller circular membrane containing all 3 hippocampal slices.
69. Pipette off the blocking solution.
70. Transfer the membranes to a 12-well plate using sterile forceps.

CRITICAL STEP: Ensure that the slices remain ‘face-up’ at all times. Use a pointed forceps to carefully transfer membranes and avoid folding of the membrane.

71. Add the appropriate concentration of primary antibodies (Table 5) in 400 µL blocking solution (i.e., PBS + 2% BSA + 0.1% Triton X) per insert.
72. Fill the empty wells with PBS to create a humidified chamber.
73. Seal the 12-well plate with parafilm and incubate at 4°C for 2 days on a platform rocker at a gentle speed.
74. After 48 h, remove the primary antibody solution.
75. Wash 3 times for 10 min with PBS.
76. Remove the final PBS solution used for washing.
77. Incubate with secondary antibodies in 400 µL of blocking solution (PBS + 2% BSA + 0.1% Triton X) per insert overnight at 4°C on a platform rocker. Ensure the secondary antibodies are incubated in the dark.
78. Remove the secondary antibody solution and wash 3 times for 10 min with PBS.
79. Using a pointed forceps, place the insert flat onto a microscope slide with the hippocampal slices facing up.
80. Add 1 drop of mounting media (non-glycerol based, e.g., ProLong Gold Antifade) and cover using a round coverslip.
81. Repeat for all insert membranes.
82. Allow the mounting media to cure for 24 h. The next day, image the hippocampal slices mounted on microscope slides using a confocal microscope (Figure 4). If slice morphology appears unhealthy or sub-optimal, refer to Table 6 for a list of potential reasons and troubleshooting tips.

**TABLE 5:** List of histological dyes and primary/secondary antibodies used for immunofluorescence, including host species, source, catalogue code and dilution factor per antibody.

Primary Antibody/Dye	Host species	Source	Catalogue code	Dilution Factor
Ki67	Rabbit	Cell signalling	d3b5	1 : 400
SOX2	Mouse	Invitrogen	MA1-014	1 : 400
Nestin	Mouse	Invitrogen	MA1-110	1 : 400
WFA	​	Invitrogen	L32481	​
AMPAR (GluA1)	Rabbit	Cell signalling	D4N9V	1 : 400
Synaptophysin	Mouse	Cell signalling	#9020	1 : 500
EdU-555	​	Invitrogen	C10638	​
NeuN	Mouse	Millipore	MAB377	1 : 500
S100β	Rabbit	Cell signalling	E7C3A	1 : 1000
DAPI	​	Sigma	MBD0015	1 : 1000
NeuroTrace™ 500/525	​	Invitrogen	N21480	1 : 300
GFAP	Chicken	Abcam	ab4674	1 : 1000

Secondary Antibody	Host species	Source	Catalogue code	Dilution Factor
anti-Rabbit 488	Donkey	Abcam	ab150061	1 : 1000
anti-Mouse 555	Donkey	Sigma	SAB4600060	1 : 1000
anti-Mouse 633	Donkey	Millipore	SAB4600131	1 : 1000
anti-Mouse 488	Donkey	Abcam	Ab15015	1 : 1000
anti-Rabbit 633	Donkey	Sigma	SAB4600132	1 : 1000
anti-Chicken 633	Donkey	Sigma	SAB4600127	1 : 1000

**TABLE 6:** Troubleshooting table for improving the growth and health of hippocampal slices.

Problem	Possible reason	Solution
Unhealthy slices	Prolonged dissection, plating, or slicing. Error in solution preparation Culturing with the cortex	Prepare and sterilise the equipment and medium before beginning dissection. Practice will decrease the time spent on each step Make a new batch of culture medium. Use fresh L-15 medium Chop off the cortex for optimal growth of the hippocampus
Bacterial or fungal contamination	Lack of sterility Contaminated medium Contaminated incubator	Make sure sterility measures and good aseptic technique are always adhered to. Sterilize, autoclave or UV all surfaces and equipment before use Discard unused medium and prepare fresh. Sterilize and clean the incubator
Incorrect slicing	Incorrect positioning of the brain tissue onto the chopping plate	If you have difficulties positioning the slice, use a spatula to flatten out the hippocampus and absorb the excess fluid with filter paper
Damaged slices during separation	Stabbing the needle into tissue during separation	Leave the cortex for slicing the brain tissue and use the area of the cortex to hold and separate the brain slices Cut off the cortex using a sterile blade after separation of the slices
Media formulation	Low growth factor concentration Sub-optimal growth or neuronal responses	Optimise the media formulation by adding growth factors, N-21 and B-27 Use of Penicillin/Streptomycin can affect neuronal function, e.g., electrophysiological parameters
Prolonged culture time	Prolonged dissection, slicing or plating	Prepare and sterilize equipment and medium before starting the experiment. Practice will decrease the time spent on each step
Dissection method	Incorrect dissection or rolling out of the hippocampus Incorrect insertion of the spatula below the corpus callosum	Develop and maintain a detailed protocol document that clearly outlines the correct steps for each procedure, including diagrams or videos illustrating the proper techniques Ask for feedback from experienced group members. Regular training sessions can help improve technique and reduce errors

**FIGURE 4:**
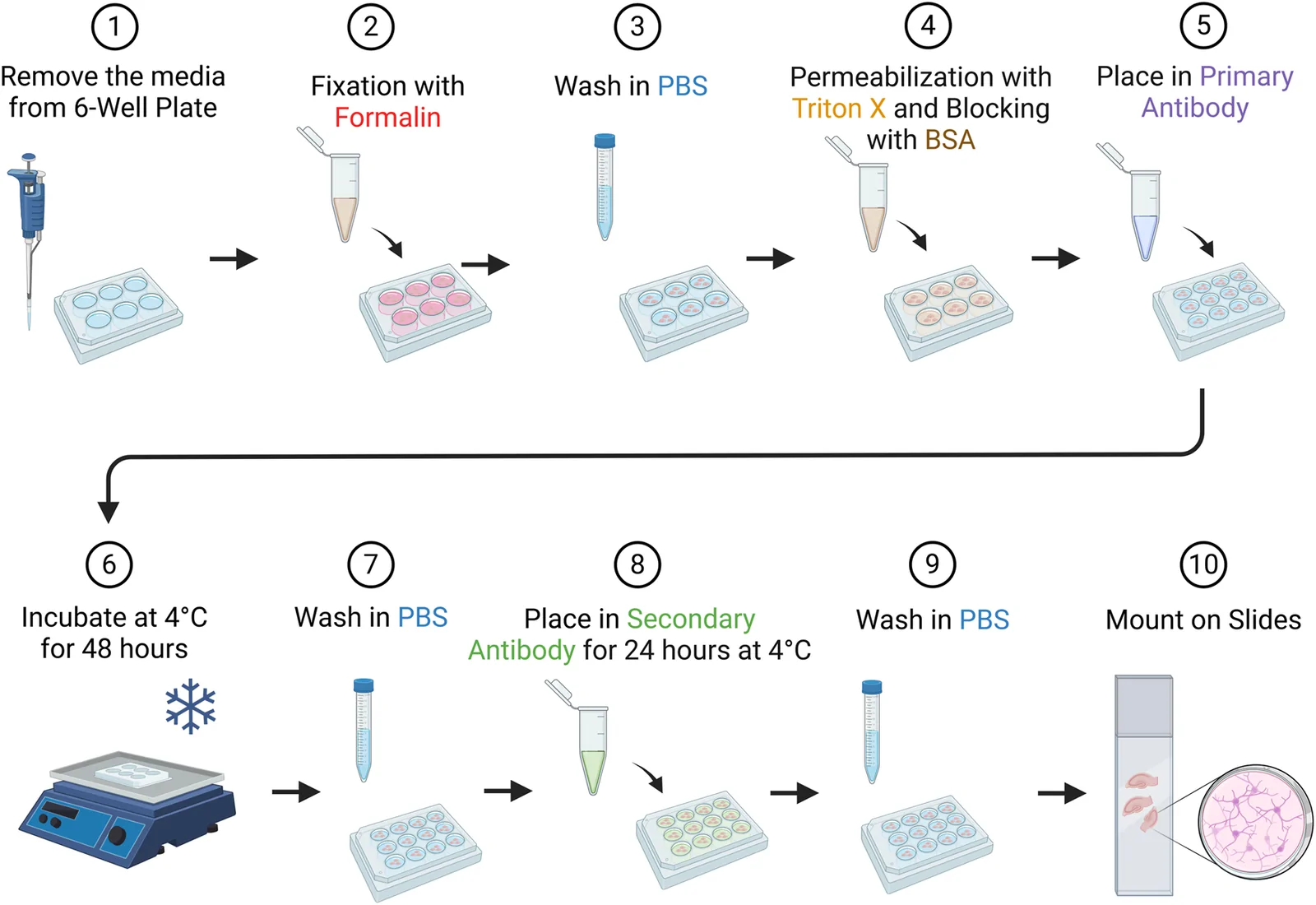
Schematic diagram describing the procedure for immunofluorescent antibody labelling of hippocampal slices. (1) Removal of the media; (2) Fixation; (3) Wash; (4) Permeabilization with Triton X and blocking with BSA; (5, 6) Incubation with primary antibodies; (7) Wash; (8) Incubation with secondary antibodies; (9) Wash; (10) Mouncaptured and the total numberting slices on microscope slides using ProLong Gold antifade mounting media. Figure created using BioRender.com.

## Results

### Characterization of organotypic hippocampal slice cultures

To validate the structural integrity and evaluate the cellular composition and neurogenic potential of our organotypic hippocampal slice cultures, we performed immunofluorescence after 14 and 19 days *in vitro* using cellular markers for proliferation, neural progenitors, and differentiated neuronal populations. Neural stem/progenitor cell populations, within the dentate gyrus (DG), were examined using antibodies targeting Ki67 and SOX2 (Figure 5). Ki67+ proliferating cells (green) were detected throughout the granule cell layers, the subgranular zone, and the Hilus indicating *in vitro* cell division. SOX2-expressing neural precursor cells (magenta) were prominently localized along the SGZ, confirming preservation of the neurogenic stem cell niche in *ex vivo* slices. Higher magnification images demonstrated that Ki67+ proliferative cells were more numerous within the Hilus and SGZ than in the outer granule cell layers of the DG, consistent with *in vivo* spatial patterns of neurogenesis.

**FIGURE 5:**
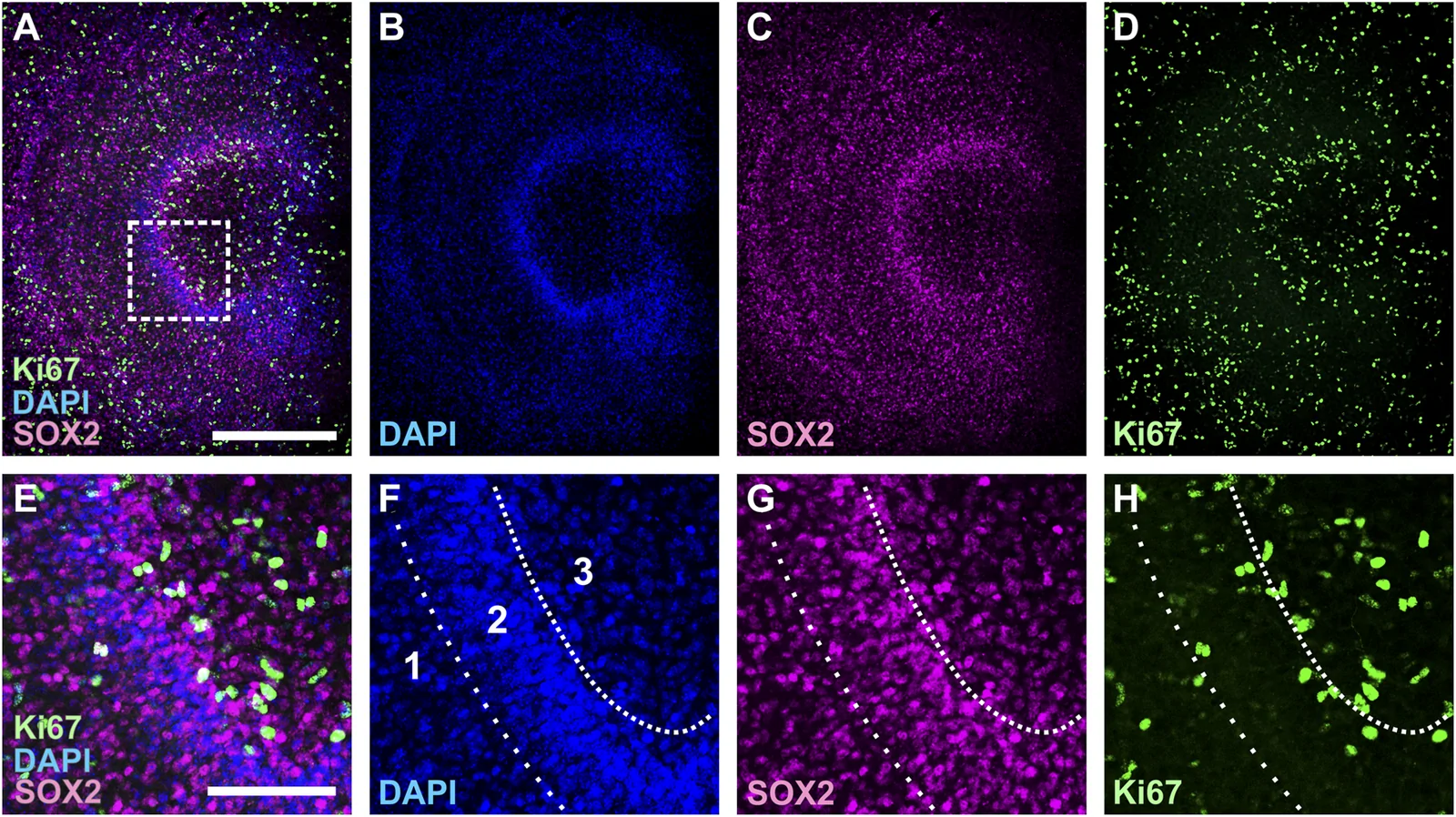
Clear delineation of the neurogenic niche in the dentate gyrus of organotypic hippocampal slices. **(A)** Immunofluorescent image of the dentate gyrus immunofluorescently-labelled with SOX2 (magenta) and Ki67 (green) antibodies and nuclei counterstained with DAPI (blue). White box represents the area captured in image panel **(E)**. Scale bar = 300 µm. **(B)** Dentate gyrus stained with DAPI nuclear marker. **(C)** Dentate gyrus labelled with SOX2 antibody. **(D)** Dentate gyrus labelled with Ki67 antibody. **(E)** Merged and zoomed image of white inset in panel **(A)**. Scale bar = 100 µm. **(F)** DAPI staining of the SGZ and neurogenic niche. 1 = outer layers of DG. 2 = neurogenic niche. 3 = Hilus. **(G)** Neurogenic niche labelled with SOX2 antibody. **(H)** Proliferative neural stem cells labelled with Ki67.

### Extracellular matrix and neural stem cell markers confirm neurogenic niches

We next evaluated the levels of extracellular matrix and the density of perineuronal nets (PNNs) in the DG using WFA (Wisteria floribunda agglutinin) fluorescein (FITC). Slices were also co-labelled with the NSC and neural progenitor cell (NPC) marker Nestin (Figure 6). PNNs regulate synaptic plasticity and neuronal excitability which impact the rate of neurogenesis [20]. WFA staining (green) revealed preserved perineuronal nets within the Hilus and dentate granule cell layers, demonstrating maintenance of key ECM components in the hippocampal slices. Nestin+ progenitor cells were abundant and retained a well-organized spatial pattern of expression within the horseshoe shape of the DG, supporting the suitability of this slice culture system for studying neural precursor cell morphology and maturation processes.

**FIGURE 6:**
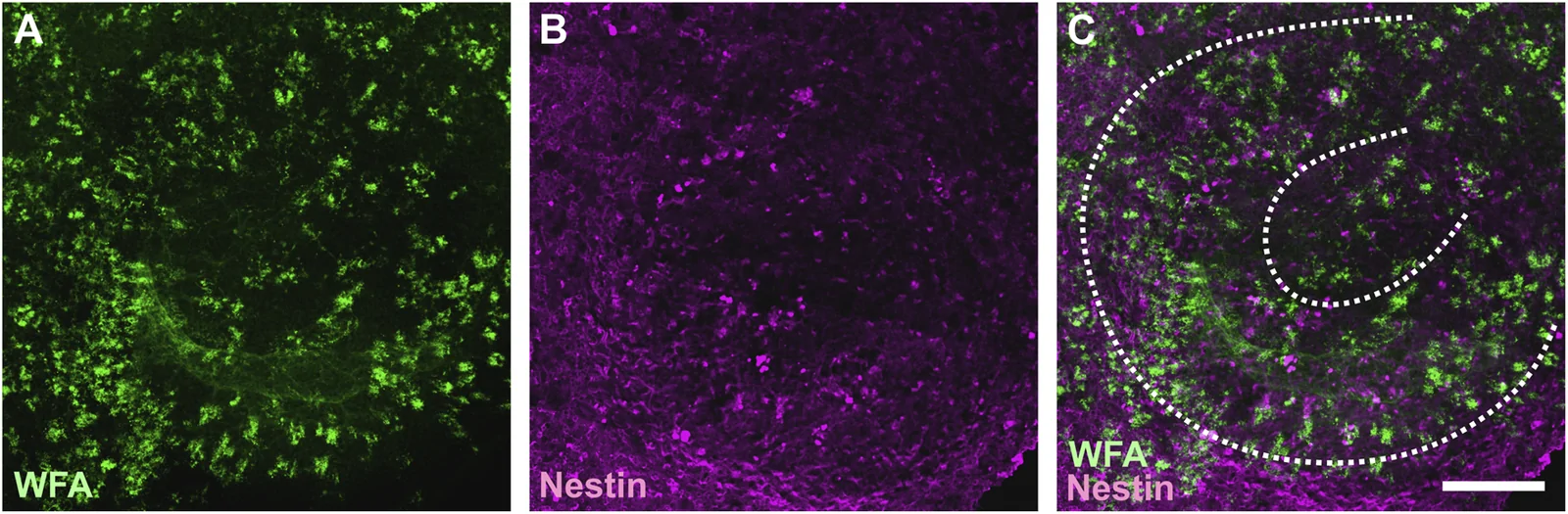
Expression of WFA and Nestin in the dentate gyrus of organotypic hippocampal slices. **(A)** Slices stained with WFA (green) fluorescein (FITC) to reveal perineuronal nets. **(B)** Slices labelled with an antibody targeting Nestin+ neural progenitor cells (magenta). **(C)** Merged image illustrating the co-localization of WFA and Nestin markers. Scale bar = 100 µm.

### Differentiated neurons are present and exhibit synaptic marker expression

To confirm the maintenance of functional neuronal circuits, particularly the presence of mossy fibre axonal tracts connecting the DG to CA3 pyramidal cells, slices were labelled for synaptophysin, a presynaptic vesicle protein, and the GluA1 subunit of the AMPA receptor, a marker of postsynaptic glutamate receptors (Figure 7). Synaptophysin expression was observed throughout the DG granule cell layer, with higher expression in mossy fibre axons projecting through the Hilus to CA3 region, indicating preserved presynaptic terminals. AMPA receptors (AMPAR) were abundantly expressed in the granule cell layer of the dentate gyrus. Merged images suggest the preservation of functional excitatory synaptic connections within cultured hippocampal slices as synaptophysin-positive axons and dendrites (green) formed dense networks throughout the slices and co-localized with AMPAR-expressing cells (magenta). These findings suggest that our organotypic slice culture system retains key cytoarchitectural features of hippocampal circuitry, facilitating the study of synaptic plasticity mechanisms alongside neurogenesis. Collectively, this immunofluorescence data demonstrate that our organotypic hippocampal slice cultures exhibit preserved *in vivo*-like cytoarchitecture with a defined SGZ niche containing a large pool of proliferative NPCs, mature extracellular matrix PNN structures in the DG, and mossy fibre-mediated connectivity between DG and CA3 regions. This characterization validates the slices as a reliable *in vitro* model for studying hippocampal neurogenesis and synaptic development.

**FIGURE 7:**
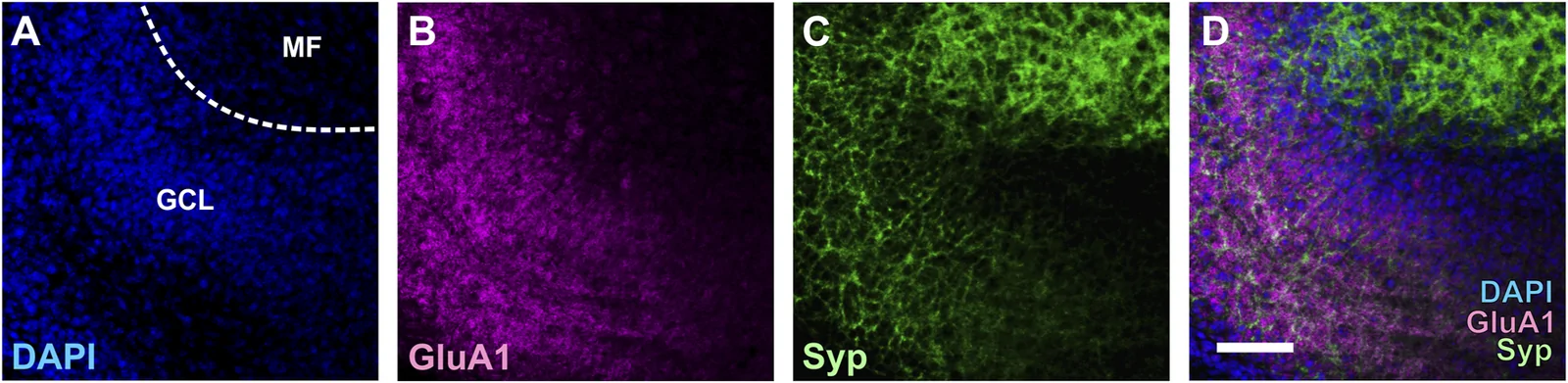
Expression of AMPA receptors (GluA1) and synaptophysin (Syp) in the dentate gyrus of organotypic hippocampal slices. **(A)** Slices stained with the cell nuclei marker, DAPI (blue). GCL = granule cell layer. MF = mossy fibres. **(B)** Slices labelled with an antibody targeting GluA1 (magenta) to reveal AMPA receptor expression. **(C)** Slices labelled with an antibody targeting synaptophysin (Syp) which marks presynaptic vesicles (green). **(D)** Merged image illustrating the co-localization of DAPI, GluA1 and synaptophysin markers. Scale bar = 50 µm.

### Assessing neural stem cell proliferation through EdU incorporation

To assess ongoing stem cell proliferation within the organotypic hippocampal slices, we performed an EdU incorporation assay coupled with NeuN and S100β immunofluorescence (Figure 8). At low magnification (×20), EdU+ nuclei (green) were distributed throughout the slice (Figures 8A–F) and represented the proportion of cells that remained mitotically active under our culture conditions. DAPI counterstaining revealed the overall cytoarchitecture of the DG, while NeuN (magenta) and S100β (red) immunolabelling distinguished mature neurons from astrocytes and oligodendrocytes, respectively.

**FIGURE 8:**
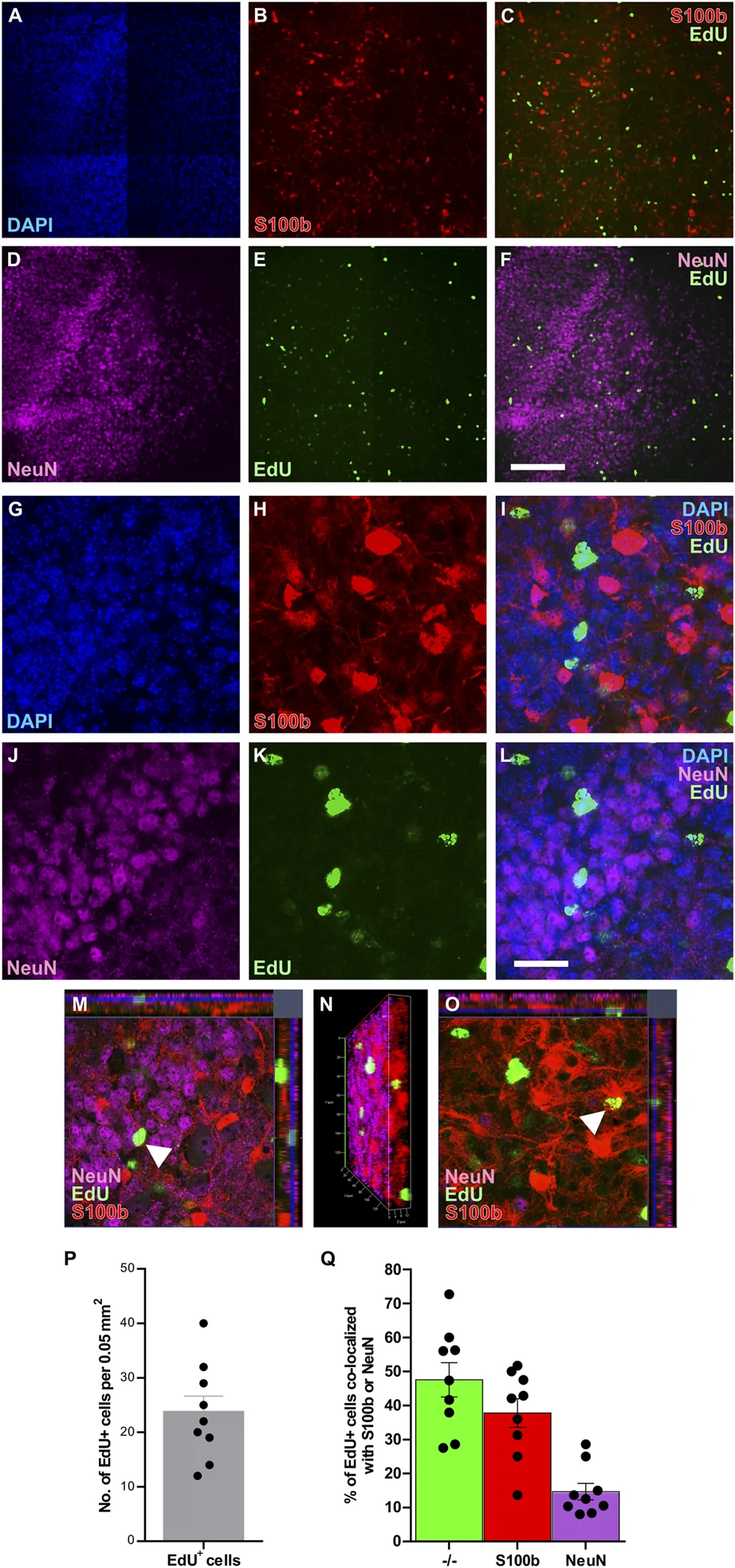
Immunofluorescence assay to assess neurogenesis and stem cell proliferation in organotypic hippocampal slice cultures. **(A–F)** Representative ×20 magnification confocal images of a hippocampal slice montaged together to capture the whole dentate gyrus. **(A)** DAPI nuclear stain (blue). **(B)** S100β labelling of astrocytes and oligodendrocytes (red). **(C)** Merged image showing the co-localization of DAPI, S100β and Edu+ cells (green). **(D)** NeuN labelling of mature neuronal cell bodies (magenta). **(E)** Proliferating cells stained with EdU (green). **(F)** Merged image showing the co-localization of DAPI, NeuN and Edu+ cells (green). Scale bar = 200 µm. **(G–L)** Representative ×63 magnification images (maximum intensity projected Z-stacks) of the granule cell layer of the dentate gyrus. **(G)** DAPI nuclear stain (blue). **(H)** S100β labelling of astrocytes and oligodendrocytes (red). **(I)** Merged image showing the co-localization of DAPI, S100β and Edu+ cells (green). **(J)** NeuN labelling of mature neuronal cell bodies (magenta). **(K)** Proliferating cells stained with EdU (green). **(L)** Merged image showing the co-localization of DAPI, NeuN and Edu+ cells (green). Scale bar = 25 µm. **(M)** Z-slice of the dentate granule neuron layer of NeuN+ cells positioned beneath the glial cell layer. White arrow highlights an EdU+ neuron representing *ex vivo* neurogenesis. **(N)** Orthogonal z-projection of the ×63 magnification image illustrating EdU+ cells in both the neuronal layer (magenta) and the glial cell layer (red). **(O)** Z-slice of the glial cell layer of S100β+ cells positioned at the surface of the slice above the deeper neuronal layer. **(P)** Mean number of EdU+ cells per 0.05 mm^2^ in the dentate gyrus (n = 9 slices). **(Q)** Percentage (%) of EdU+ cells in the dentate gyrus co-localized with either S100β, NeuN or neither (−/−).

We quantified the mean density of EdU+ cells in each dentate gyrus. Three ×20 magnification confocal images of the granule cell layers were captured and the total number of EdU+ cells per image were counted. We calculated an average of 24 (±9) EdU+ cells per 0.05 mm^2^ in the DG region (Figure 8P). This result illustrates that the slice culture system maintains a consistent and reliable proliferative capacity, further validating its use as a platform for studying postnatal neurogenesis in an *ex vivo* hippocampal environment.

Next, we captured ×63 magnification z-stack images of the DG (Figures 8G–L). As expected, NeuN-positive neuronal cell bodies were densely expressed in the dentate granule cell layer, whereas S100β-expressing glia were more dispersed throughout the subregions of the DG. Merged images demonstrated that EdU incorporation occurred in both neuronal and non-neuronal regions. Interestingly, orthogonal z-projections of the hippocampal slice illustrate that a layer of glial cells (red S100β+ cells) sits above the dentate granule neuronal layer (magenta NeuN+ cells) (Figure 8N). Moreover, EdU+ cells are present in both neuronal (Figure 8M) and glial cell layers (Figure 8O) as indicated by the white arrows. To quantify the cell types expressing EdU, we calculated the proportion of EdU+ cells expressing either NeuN, S100β or neither (−/−) markers (Figure 8Q).

S100β+ and EdU+ cells represented approximately 35–40% of the proliferative cell population. NeuN+ and EdU+ cells represented approximately 15% of the proliferative population. Finally, EdU+ cells that were double-negative (−/−) for S100β and NeuN+ made up approximately 45–50% of the proliferative cell population. These results illustrate the presence of a neural stem cell pool in the DG as well as NPCs that have actively differentiated into either astrocytes and oligodendrocytes (40%) or into mature post-mitotic neurons (15%).

## Discussion

To preserve the cytoarchitecture of the hippocampal formation and neurogenic niche *ex vivo*, it is essential to protect not just the survival of individual cells, but the delicate spatial arrangement of subregions, ECM, neurons, glia, white matter and axonal projections that give the structure its functional identity. While the membrane-interface technique described by Stoppini et al. [12] provided a robust platform for long-term cultivation of 350–400 µm brain slices, the original medium formulation relied on a high-serum environment that often favoured glial proliferation at the expense of the neurogenic niche. The current protocol represents a significant refinement of this method, utilizing a tiered media transition and a sophisticated biochemical milieu to sustain the delicate balance of hippocampal neurogenesis and synaptic maturation. However, an increase in glial cell reactivity caused by tissue slicing is inevitable in *ex vivo* organotypic preparations.

### Refinement and optimisation of the culture milieu: from high serum to defined supplements

Our protocol diverges from the traditional Stoppini formulation by implementing a phased transition from a 10% serum-based medium plus growth factors (NGF, EGF, FGF) to a 5% serum-containing medium with growth factors and defined supplements (B27 and N21). This acknowledges the shifting metabolic and trophic requirements of the hippocampal slice during the first few days of the recovery period to the maintenance phase for long-term cultures. Early studies utilized high concentrations of horse serum (up to 25%) to facilitate tissue flattening and initial survival [11, 12]. However, unrefined serum contains an unpredictable array of factors that varies lot-to-lot and by source and can enhance reactive gliosis, thus inhibiting the sensitive developmental mechanisms that drive NPC proliferation, migration and differentiation into functional neurons. To mitigate this reactive glial phenotype, more recent protocols have successfully pivoted toward chemically defined serum-free media. For instance, Mayerl and ffrench-Constant demonstrated that supplementing Neurobasal/B-27 media with the anti-inflammatory compound, indomethacin, directly dampens astrogliosis and actively preserves the *ex vivo* neurogenic potential of the hippocampal niche [21].

In our approach, the initial recovery phase utilizes a balanced combination of 6% horse serum (HS) and 4% foetal bovine serum (FBS). This pairing exploits the complementary properties of these additives, i.e., the mitogenic richness of FBS, laden with platelet-derived growth factor (PDGF) and fibroblast growth factors (FGF) which support the immediate proliferative needs of the subgranular zone (SGZ), while the milder profile of HS facilitates tissue stabilization without triggering excessive excitotoxicity. We have previously implemented a media switch on day 3 from 25% serum-containing salt solution to Neurobasal-A with B27-supplemented media and 0% serum for growing cerebellar slice cultures [22, 23]. However, we found that hippocampal slices were more sensitive to the abrupt change from high serum to a zero serum defined medium. As such, dropping the initial serum content to 10% followed by 7.5% on days 3–5 and finally to 5% from day 5 onwards, ensured the slices experienced a milder phased transition to a low serum environment.

The inclusion of MITO+ serum extender to both media #1 and #2 provides a concentrated suite of hormones and growth factors, including EGF, FGF, insulin, transferrin, and selenium (ITS), alongside progesterone and putrescine. This cocktail aims to maintain the necessary mitogenic stimuli for neural stem cells whilst facilitating a significant drop in serum concentration. This is particularly relevant given the work of Raineteau et al. [24] who showed that chronic EGF exposure can increase neurogenesis in organotypic hippocampal cultures but that horse serum (25%) can abolish this effect. Moreover, Namba et al. [25], demonstrated that while spontaneous neurogenesis occurs in organotypic slices, the neurogenic capacity of the dentate gyrus progressively declines *in vitro*, likely due to loss of niche signals and reactive changes, highlighting the need for defined culture conditions to sustain progenitor proliferation. By providing a defined mitogenic stimulus via MITO+ and nerve growth factor (NGF), we extend the window of active neurogenesis well into the third week *in vitro*. These findings mirror previous efforts to control neurogenesis in the subgranular zone niche using defined chemical additives. Previous studies have shown that the endogenous progenitor pool within postnatal slice cultures remains highly responsive to external chemical cues. For instance, Poulsen et al. [26] demonstrated that targeted media supplementation with growth factors (EGF and IGF-I) as well as small-molecule glutamate receptor antagonists such as NMDA blockers (MK-801 and APV) or the AMPA antagonist, CNQX, significantly increases the differentiation of pre-existing neural precursor cells into TUC-4+ dentate granule cells. Interestingly, these small-molecule inhibitors reduced baseline glutamatergic tone which synergises with biochemical mitogens to guide precursor cell fate without inducing cytotoxicity. By utilizing an optimized basal medium containing MITO+ serum extender and NGF, our protocol similarly harnesses this inherent plasticity and directs neuronal maturation, establishing a controlled baseline that can be easily combined with small-molecule modulators of neurogenesis.

### Metabolic refinement and antioxidant protection

Organotypic hippocampal slices are often grown in culture at sub-physiological temperatures between 35 and 36 °C [9, 12, 17]. Studies have shown that mild hypothermia is neuroprotective [27, 28] and thus we maintained slices at a constant temperature of 35.5 °C for the duration of the culture. The transition at day 3 to a Neurobasal-A backbone, supplemented with B27 and N21, represents a move toward a more physiological maintenance phase. Neurobasal-A, optimized for the survival of post-mitotic neurons, lacks the high glutamate levels found in standard basal media, thereby reducing chronic excitotoxicity [19]. The B27 supplement is essential for its antioxidant properties; it contains catalase, superoxide dismutase, and vitamin E, which mitigate the oxidative stress inherent in high-oxygen interface cultures. Furthermore, the addition of N21 (also known as NS21) provides a more defined and consistent source of essential fatty acids and vitamins compared to original B27 lots. Research by De Simoni et al. [29] underscored the importance of such defined media for the development of CA1 pyramidal neurons and the formation of mature dendritic spines. Our observation of high synaptophysin and AMPA receptor expression suggests that this supplemented environment supports not only the birth of new neurons but their successful integration into functional, mature synaptic networks.

### The neurogenic niche and lineage commitment

Our immunofluorescent results quantitatively validate the fidelity of this slice culture system. The presence of dense Ki67 and SOX2 clusters within the SGZ, alongside high levels of Nestin, confirms that the primary neurogenic reservoir remains localized and active. This spatial restriction is critical; as Namba et al. [25] noted, the loss of neurogenic potential in many slice models is often preceded by the dispersal of these progenitor populations.

Our EdU lineage tracing data reveals the following differentiation profile:

- 15% NeuN+ (neurons): Representing the terminal differentiation of progenitors into a neuronal lineage, a process fundamentally guided by the local hippocampal environment.
- 40% S100β+ (astrocytes and oligodendrocytes): Reflecting the ongoing generation of macroglia required to maintain the ionic and metabolic stability of the slice.
- 45% double-negative (−/−): This substantial population likely comprises the transit-amplifying progenitors (Type 2 cells), which provide the continuous proliferative capacity of the DG.

The preservation of the extracellular matrix (ECM), evidenced by robust Wisteria floribunda agglutinin (WFA) staining for perineuronal nets (PNNs), is perhaps the most significant advantage of this 3D system. PNNs are critical for stabilizing the synaptic architecture and defining the boundaries of the neurogenic microenvironment. In the absence of these structures, as is often seen in 2D monolayer cultures or emergent organoid models, the mechanical and biochemical cues required for directed neurogenesis are lost.

### Limitations: structural remodelling and reactive gliosis in slice cultures

Although organotypic slice cultures retain the principal cytoarchitecture of the hippocampal formation, tissue preparation necessarily involves axotomy of major afferent and efferent projections. Removal of the entorhinal cortex abolishes perforant pathway input to the dentate gyrus, while tissue sectioning transects commissural and associational fibres that normally provide recurrent and interhemispheric connectivity. As a result, the trisynaptic hippocampal circuit is effectively deafferented and functionally isolated from key extrinsic inputs. This loss of connectivity triggers reactive synaptogenesis and substantial network reorganization, including mossy fibre sprouting into the inner molecular layer of the dentate gyrus and remodelling of recurrent CA3 collateral circuitry [29–32]. These anatomical adaptations increase recurrent excitation and reshape network dynamics, progressively promoting hyperexcitability and lowering the threshold for epileptiform activity [33].

To mitigate these circuit-level artefacts and better preserve physiological patterns of connectivity, several complementary approaches can be adopted. Retention of entorhinal cortex input (Figure 1B) through organotypic entorhino-hippocampal co-cultures preserves perforant pathway connectivity and reduces the extent of deafferentation imposed by conventional hippocampal slice preparation [34, 35]. By maintaining this major afferent projection, these slice preparations provide a closer approximation of native hippocampal network architecture. However, we have found that hippocampal slices, particularly the dentate gyrus, appear to survive better without the neocortex and entorhinal cortex attached. Pharmacological strategies may also be used to limit excessive network activity; however, glutamatergic signalling is itself a key regulator of injury-induced circuit plasticity. Consequently, glutamate receptor antagonism modifies rather than prevents reactive remodelling. For example, blockade of NMDA receptors promotes axonal sprouting, whereas signalling through AMPA/kainate receptors is required for reactive sprouting and the recovery of functional connectivity following deafferentation [36, 37]. Any intervention targeting glutamatergic transmission may therefore alter the trajectory of circuit reorganization rather than restore baseline excitability of the native hippocampal network.

A further limitation inherent to organotypic slice cultures is the mechanical trauma of tissue sectioning, which initiates inflammatory and glial responses characteristic of long-term culture adaptation. The magnitude of this tissue injury is influenced by the slicing method. The McIlwain tissue chopper enables rapid and highly reproducible preparation of large numbers of slices and therefore remains widely used for organotypic culture protocols. In contrast, vibratome sectioning reduces tissue compression and shear forces during cutting, resulting in improved preservation of tissue architecture at the slice surface and enhanced morphological integrity of the preparation [18, 38]. However, these advantages must be balanced against longer preparation times. Vibratome sectioning is considerably slower and extends the interval between tissue harvest and culture, increasing exposure to *ex vivo* metabolic stress. In contrast, the speed of tissue chopping minimises this period and may facilitate more rapid recovery of cellular metabolism following culture establishment. Regardless of the preparation method used, tissue sectioning induces a robust reactive glial response. Consistent with this, our immunofluorescence analysis revealed pronounced GFAP-positive astrogliosis in cultured hippocampal slices (Figure 9). Importantly, reactive gliosis and neuroinflammatory signalling are persistent features of long-term organotypic cultures, reflecting the combined effects of axotomy, deafferentation, and adaptation to the *in vitro* environment [39, 40]. Consequently, selection of a slicing strategy involves a practical trade-off between minimising acute mechanical damage and minimising preparation-associated metabolic stress.

**FIGURE 9:**
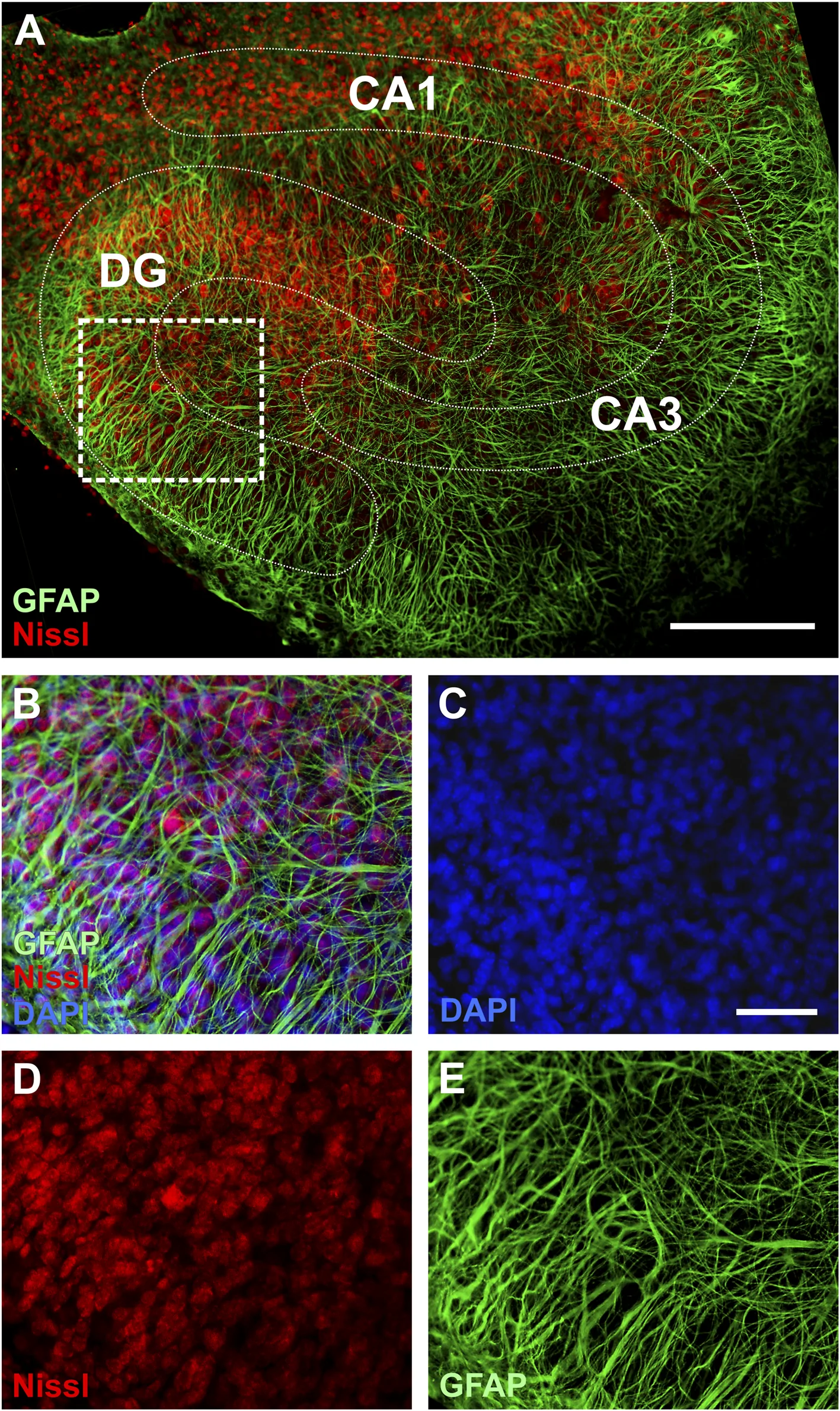
Reactive astrogliosis in the dentate gyrus of organotypic hippocampal slices. **(A)** Two-week old slice labelled with GFAP (green) to highlight the extensive astrocyte syncytium present in hippocampal cultures and with Nissl (red) to illustrate neuronal nuclei. DG = dentate gyrus. CA3 = Cornu Ammonis area 3. CA1 = Cornu Ammonis area 1. Scale bar = 200 µm. **(B)** Zoomed image of the dentate granule cell layer represented by the white square inset region in image **(A)**. **(C)** Cell nuclei stained with DAPI (blue). Scale bar = 50 µm. **(D)** Neuronal nuclei stained with Nissl (red). **(E)** Astrocytes immunofluorescently-labelled with a GFAP antibody (green).

The organotypic hippocampal slice culture protocol presented here is also compatible with emerging three-dimensional imaging and tissue-processing approaches. Organotypic slice cultures can be combined with tissue-clearing and refractive index matching methods, such as ScaleS, as well as expansion microscopy (ExM) and light-sheet fluorescence microscopy (LSFM), to improve imaging depth and visualization in thick tissue preparations [41–43]. For neurogenesis studies, LSFM may facilitate long-term, low-phototoxicity imaging of progenitor cell behaviour, enabling tracking of cell division, migration and neuronal integration over time. In parallel, ExM enables nanoscale-resolution visualization of protein distributions and subcellular structures within intact tissue, including synaptic receptor localization and microcircuit organization [43, 44]. Together, these complementary approaches could support high-resolution volumetric analyses of lineage progression, neuronal maturation and circuit integration in hippocampal slice cultures *ex vivo*.

### Advantages over iPSC-derived organoids

While human iPSC-derived organoids have become a prominent feature of modern neuroscience, they frequently lack the structural maturity and laminar precision found in our rodent organotypic slices. Organoids often suffer from stochastic cell placement and the absence of a defined vascular-like scaffold or a mature, organized ECM. Specifically, the mossy fibre pathway and the precise orientation of the radial glial scaffold, which are both pre-assembled in our slice model, are essential for the authentic integration of new neurons into the tri-synaptic circuit. As we move toward a more comprehensive understanding of neurodevelopmental disorders, the preservation of this functional anatomy becomes paramount. Our refined protocol, by honouring the structural template of the hippocampus while modernizing the biochemical support system, offers a high-fidelity platform for long-term neurogenesis assays. It bridges the gap between simple monolayer cell cultures and the complexity and inaccessibility of the hippocampus during *in vivo* studies.

Arguably, the most appropriate *ex vivo* system to investigate dentate granule cell physiology is human organotypic hippocampal slice cultures which have been generated by several research groups [45, 46]. These valuable research tools bridge the gap between iPSC-derived brain organoids and *in vivo* functional imaging techniques, but the excised samples of human brain tissue are extremely rare, lack consistency and are often biopsied from patients with brain tumours or epilepsy, for example, [47]. For most basic neuroscientists without neurosurgical collaborators; if the precise anatomical structure of the hippocampal circuitry and SGZ niche cytoarchitecture remains central to an investigation, then rodent organotypic brain slices are valuable models for interrogating the synaptic plasticity and functional maturation of hippocampal neural networks.

## Data Availability

The original contributions presented in the study are included in the article/supplementary material, further inquiries can be directed to the corresponding author.

## Glossary

AMPAR: α-amino-3-hydroxy-5-methyl-4-isoxazolepropionic acid receptor
B27: Serum-free neuronal culture supplement B27
BSA: Bovine serum albumin
CA1: Cornu Ammonis area 1 of the hippocampus
CA3: Cornu Ammonis area 3 of the hippocampus
**CO**_**2**_: Carbon dioxide
CSF: Cerebrospinal fluid
DAPI: 4′,6-diamidino-2-phenylindole
DG: Dentate gyrus
DIV: Days *in vitro*
DMEM/F12: Dulbecco’s Modified Eagle Medium/Ham’s F-12
DMSO: Dimethyl sulfoxide
ECGS: Endothelial cell growth supplement
ECM: Extracellular matrix
EdU: 5-ethynyl-2′-deoxyuridine
EGF: Epidermal growth factor
FBS: Foetal bovine serum
FGF: Fibroblast growth factor
FITC: Fluorescein isothiocyanate
HBSS: Hank’s balanced salt solution
HEPES: 4-(2-hydroxyethyl)-1-piperazineethanesulfonic acid
HS: Horse serum
IGF-1: Insulin-like growth factor 1
Ig/IgG: Immunoglobulin/Immunoglobulin G
iPSC: Induced pluripotent stem cell
ITS: Insulin–transferrin–selenium
Ki67: Cellular marker of proliferation
L-15: Leibovitz’s L-15 medium
mM: Millimolar
mOsm/L: Milliosmoles per litre
NGF: Nerve growth factor
NeuN: Neuronal nuclei (RBFOX3), marker of mature neurons
Neurobasal-A: Neuronal basal medium optimized for post-mitotic neurons
N21/NS21: Defined neuronal supplement (optimized alternative to B27)
NPC: Neural progenitor cell
NSC: Neural stem cell
OHSC: Organotypic hippocampal slice cultures
PBS: Phosphate-buffered saline
PDGF: Platelet-derived growth factor
PNN: Perineuronal net
PHN: Postnatal hippocampal neurogenesis
PPE: Personal protective equipment
ROS: Reactive oxygen species
SGZ: Subgranular zone
SOX2: SRY-box transcription factor 2
S100β: S100 calcium-binding protein beta
Syp: Synaptophysin
T3: Triiodothyronine
WFA: Wisteria floribunda agglutinin
Z-stack: Series of optical sections collected along the z-axis
